# Rhomboid intercostal block combined with sub-serratus plane block versus rhomboid intercostal block for postoperative analgesia after video-assisted thoracoscopic surgery: a prospective randomized-controlled trial

**DOI:** 10.1186/s12890-021-01432-7

**Published:** 2021-02-25

**Authors:** Wei Deng, Xiao-min Hou, Xu-yan Zhou, Qing-he Zhou

**Affiliations:** Department of Anesthesiology and Pain Medicine, The Affiliated Hospital of Jiaxing University, Zhejiang Province, Jiaxing, China

**Keywords:** Rhomboid intercostal block, Serratus plane block, Video-assisted thoracoscopic surgery

## Abstract

**Background:**

Rhomboid intercostal block (RIB) and Rhomboid intercostal block with sub-serratus plane block (RISS) are the two types of plane blocks used for postoperative analgesia after video-assisted thoracoscopic surgery (VATS). This prospective randomized controlled trial was performed to analyze the postoperative analgesic effects of ultrasound-guided RIB block and RISS block after video-assisted thoracoscopic surgery.

**Methods:**

Ninety patients aged between 18 and 80 years, with American Society of Anesthesiologists physical status Classes I–II and scheduled for elective unilateral VATS were randomly allocated into three groups. In group C, no block intervention was performed. Patients in group RIB received ultrasound-guided RIB with 20-mL 0.375% ropivacaine and those in group RISS received ultrasound-guided RIB and serratus plane block using a total of 40-mL 0.375% ropivacaine. All patients received intravenous sufentanil patient-controlled analgesia upon arrival in the recovery room. Postoperative sufentanil consumption and pain scores were compared among the groups.

**Results:**

The dosages of sufentanil consumption at 24 h after the surgery in the RIB and RISS groups were significantly lower than that in group C (*p* < 0.001 and *p* < 0.001 for all comparisons, respectively), the postoperative Numerical Rating Scale (NRS) scores in the RIB and RISS groups at 0.5, 1, 3, 6, 12, 18, and 24 h after surgery when patients were at rest or active were significantly lower than that in group C (*p* < 0.05 for all comparisons). The required dosage of sufentanil and time to first postoperative analgesic request in groupRISS were less than those in the group RIB at 24 h after the surgery (*p* < 0.001 and *p* < 0.001 for all comparisons, respectively). Similarly, the Numerical Rating Scale scores for group RISS at 12, 18, and 24 h after the surgery when the patients were active were significantly lower than those for group RIB (*p* < 0.05 for all comparisons).

**Conclusion:**

Both ultrasound-guided RIB block and RISS block can effectively reduce the demand for sufentanil within 24 h after VATS, and less sufentanil dosage is needed in patient with RISS block. Ultrasound-guided RIB block and RISS block can effectively relieve pain within 24 h after VATS, and RISS block is more effective.

## Introduction

Postoperative pain is a significant concern following video-assisted thoracoscopic surgery (VATS) [1, 2]. Pain after thoracic surgery not only causes a strong stress reaction and adverse emotional experience but also affects postoperative rehabilitation [3–5]. Hence, different analgesia techniques, including local anesthetic infiltration, intercostal nerve block, paravertebral block, and thoracic epidural anesthesia, have been described to attenuate the intensity of acute postoperative pain [6, 7]. However, the analgesic effects of local anesthetic infiltration and intercostal nerve block are short and poor [8]. Paraspinal block and thoracic epidural anesthesia may cause parasympathetic symptoms, resulting in hypotension, bradycardia, and even syncope [7]. Additionally, they could lead to general spinal anesthesia, local hematoma, infection, anesthetic poisoning, and paraspinal muscle pain [9, 10].

Recently, some studies have shown that the rhomboid intercostal block (RIB) and the RIB combined with the sub-serratus plane block (RISS) can provide good analgesia effects after VATS [11–14]. The ultrasound-guided RIB and RISS blocks are two novel analgesic techniques recently described by Elsharkawy et al. [15, 16]. Additionally, the RISS block anesthetizes the lateral cutaneous branches of the thoracic intercostal nerves and can be used in multiple clinical settings for chest wall and upper abdominal analgesia [15]. However, the analgesic effects of the RISS block after VATS have not been analyzed through a randomized-controlled trial.

This prospective randomized controlled trial was performed to analyze the postoperative analgesic effects of ultrasound-guided RIB block and RISS block after video-assisted thoracoscopic surgery. The primary hypothesis of this study is that the ultrasound-guided RISS block reduced postoperative sufentanil consumption and pain scores more effectively than the RIB in the first 24 h after VATS.

## Methods

### Ethics

The study was designed as a single-center, prospective, randomized-controlled trial. Ethical approval for this study (Ethics Committee of the Affiliated Hospital of Jiaxing University) was provided by the Ethics Committee of the Affiliated Hospital of Jiaxing University (LS2020-298), Jiaxing, China (Chairperson Prof Qh Zhou) on August 1, 2020. This study was conducted following the principles outlined in the Declaration of Helsinki. The study protocol was registered in the Chinese Clinical Trial Register (ChiCTR2000038264, links to registration documents: http://www.chictr.org.cn/showprojen.aspx?proj=59083), The Chinese Clinical Trial Registration date is September 15, 2020 (15/09/2020), and the date of patient enrollment is September 17, 2020 (17/09/2020). All participants provided written informed consent before study enrollment.

### Participants and design

Written informed consent, both for the interventions and enrollment in the study, was obtained from all participants. Patients aged between 18 and 80 years, with American Society of Anesthesiologists (ASA) physical status Classes I and II, and scheduled for elective unilateral VATS were screened for enrollment in the study. The operation was expected to be completed at 14:00 every day. The exclusion criteria were patients with shock or coma, abnormal blood coagulation, infection in the planned area of block, severe nerve injury on the side of the limb, history of chronic pain requiring analgesics, psychiatric diseases, radiotherapy, chemotherapy, contraindications to non-steroidal anti-inflammatory drugs, allergy of local anesthetic drugs such as lidocaine and ropivacaine or of general anesthetics, who refused surgical anesthesia, history of previous mastectomy or other thoracic surgery, body mass index (BMI) ≥ 35 kg/m^2^, and inability to use patient-controlled analgesia (PCA).

### Anesthesia application

After shifting to the preoperative area, all patients underwent conventional monitoring procedures including electrocardiography, noninvasive monitoring of blood pressure, and peripheral oxygen saturation measurements. Intravenous access was gained using a 22-gauge intravenous needle, and isotonic saline was infused at a rate of 15 mL kg^−1^ h^−1^. Anesthetic management was in accordance with a standard protocol. Anesthesia was induced with pre-oxygenation for 3 min followed by intravenous injection of midazolam (0.05 mg/kg), sufentanil (0.5 µg/kg), propofol (1–2 mg/kg), and cisatracurium (0.15 mg/kg). A double-lumen endotracheal catheter was used for positive-pressure ventilation to maintain the end-tidal carbon dioxide level of 35–40 mmHg.

Anesthesia was maintained using 2% sevoflurane with 50% oxygen, remifentanil (0.5 µg kg^−1^ min^−1^), and propofol (100 µg kg^−1^ min^−1^). Additionally, cisatracurium (0.15 mg/kg) was administered according to the surgical protocol. Surgery (Unilateral thoracoscopic lobectomy) was implemented via a single 3.0–4.0-cm incision in the fourth or fifth intercostal space of the anterior axillary line as the operation hole by the same surgeon group. The anesthetic dose was adjusted to maintain blood pressure within 20% of the baseline value. An additional dose of intravenous remifentanil (0.1–1.0 µg kg^−1^ min^−1^) was injected as needed. If the blood pressure decreased by > 20% from the baseline value, 250 mL of 0.9% (physiologic) saline and ephedrine (0.1 mg/kg) were administered. If the heart rate decreased to less than 50 bpm, atropine (0.5 mg/kg) was administered [15]. At the end of the VATS, the effect of cisatracurium was reversed using neostigmine and atropine as needed [7]. After the surgery, patients were transferred to the postoperative recovery room, where the endotracheal tube was removed. All patients received intravenous granisetron 3 mg upon arrival in the recovery room.

### Patient grouping and randomization

After endotracheal intubation, the patients were randomly allocated into three groups based on a computerized randomization table created by a researcher who was not involved in the study. The researcher assigned a random ID to each patient, and a blinded anesthesiologist used this ID while collecting the postoperative data in the surgical ward [9].

### Application of block intervention

Following endotracheal intubation, patients allotted to the RIB group were positioned in the lateral decubitus position with the chest on the operating side lying superiorly. The ipsilateral arm was abducted from the chest to move the scapula laterally. The RIB was performed as described previously [8]. A high-frequency (6–12 MHz) linear ultrasound probe (LOGIQ e ultrasonic system, Deutschland GmbH & Co. KG, Solingen, Germany) was placed medial to the medial border of the scapula in the oblique sagittal plane. The landmarks, i.e., the trapezius muscle, rhomboid muscle, intercostal muscles, pleura, and lung, were identified in the ultrasound. Under aseptic conditions, an 80-mm 21-gauge needle was inserted at the level of T5–6 in the ultrasound view. A single dose of 20-mL 0.375% ropivacaine was injected in the interfascial plane between the rhomboid major and intercostal muscles. The spread of the local anesthetic solution under the rhomboid muscle was visualized by ultrasonography.

In patients allotted to the RISS group, a linear ultrasound probe was placed in the sagittal plane at the T5–6 level, just medial to the scapula, to identify the trapezius, rhomboid major, and intercostal muscles. A 21-gauge needle was inserted in the plane between the rhomboid major and intercostal muscles in a cephalad to caudad direction, and 20 ml of 0.375% ropivacaine was injected [8]. Thereafter, the ultrasound probe was moved caudally and laterally to identify the tissue plane between the serratus anterior and external intercostal muscles for the sub-serratus block at the T8–9 level. The needle was advanced from its previous position, and an additional 20 mL of 0.375% ropivacaine was injected [12, 13]. All block procedures were performed by the same anesthesiologist who had administered the RIB and RISS blocks in more than 30 cases before this study. In control group (group C), no block intervention was performed.

### Analgesic protocol and evaluation of pain and sensorial block

In the post-anesthesia care unit, all patients received patient-controlled intravenous analgesia (PCIA): 100 μg sufentanil with a total of 100 mL, background dose of 2 mL, self-administered bolus dose of 1.5 mL, and locking time of 20 min. Another blinded anesthesiologist conducted pain assessments at the postoperative 30th min by using the 11-point Numerical Rating Scale (NRS), which ranges from ‘0’ (no pain) to ‘10’ (worst pain imaginable). The patients were transferred to the surgical ward at the end of the 30th min. In the surgical ward, the patients were assessed again at 0.5, 1, 3, 6, 12, 18, and 24 h, postoperatively. If the postoperative NRS score was greater than 3, the analgesia pump was pressed once, and the pain was evaluated after 30 min. If the NRS score continued to be greater than 3, the analgesia pump was pressed again.

### Outcome measures

The primary outcome measures were total postoperative sufentanil consumption and NRS scores of patients at different time points in the first 24 h. The secondary outcome measures were the doses of remifentanil and propofol, time to first postoperative analgesic request, and satisfaction score of patients (1–10, whereby 10 is the highest). In addition to these measures, postoperative nausea and vomiting (PONV, which were rated on a four-point verbal scale: none = no nausea, mild = nausea but no vomiting, moderate = vomiting one attack, severe = vomiting > one attack), and block-related complications such as pneumothorax, bleeding, allergy, and local anesthetic toxicity were also recorded.

### Sample size

The sample size for this study was calculated using PASS 15 (PASS 15.0 is a powerful sample size and power software of choice for clinical trials, pharmaceuticals and other medical research. It also serves as a pillar for all other areas that require sample size calculation or evaluation.) based on a pilot study with 10 patients in each group. The mean sufentanil consumptions in 24 h were 61.8 ± 6.0 μg in group C, 52.2 ± 4.0 μg in the RIB group, and 51.5 ± 3.5 μg in the RISS group. Assuming an α error of 0.01 (two-tailed) with a power of 0.90, at least 24 participants were needed per group. Considering the potential patient dropout rate, we decided to include 30 patients in each group.

### Statistical analysis

Statistical analyses were performed using SPSS v25.0 (IBM, Armonk, NY, USA). The distributions of variables in this study were assessed using the Shapiro–Wilk test, whether the observations were normal or skewed. When the data were normally distributed, they were presented as mean ± standard deviation. Continuous data that yielded non-parametric dispersion were presented as median and interquartile range and were analyzed using the Mann–Whitney U test to assess the differences between groups. One-way analysis of variance (ANOVA) was used to compare the differences in outcome parameters (age, BMI, duration of procedure, duration of anesthesia, remifentanil dose, propofol dose, recovery time, NRS score, NRS dynamic score, time to first postoperative analgesic request, total sufentanil consumption in 24 h, and satisfaction scores) among groups. An independent sample *t* test was used to compare the differences in outcome parameters (remifentanil dose, propofol dose and satisfaction scores) among RIB and RISS groups. The prevalences of nausea, vomiting, ASA I/II, hypertension, diabetes, surgical incision (location of surgical incision in patients), and limb paresthesia were presented as percentages, and the differences among groups were evaluated using the chi-square test. Pairwise comparison among groups for one-way ANOVA was performed using post-hoc analysis and the Student–Newman–Keuls Q-test. The corrected *p* value was obtained directly, and the cutoff value was 0.05.

## Results

**Fig. 1:**
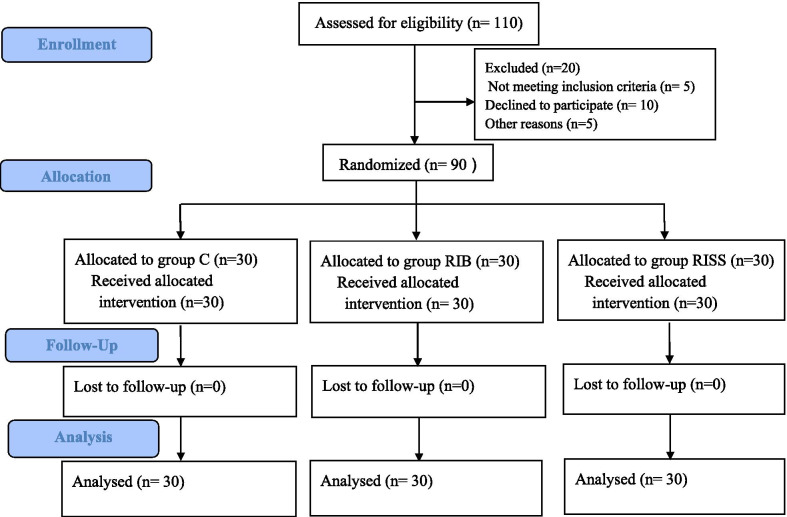
Consolidated Standards of Reporting Trials (CONSORT) flow diagram for the study

One hundred and ten patients were screened to be eligible for inclusion in this study; 20 patients were excluded, of which 4 patients did not meet the inclusion criteria, 12 patients refused to participate in this study, and 4 patients were not allotted to any experimental group for other reasons. Finally, 90 patients were equally allocated to the 3 study groups. The Consolidated Standards of Reporting Trials (CONSORT) diagram for the study is shown in Fig. 1. There was no significant difference in demographic characteristics among the three treatment groups (see Table 1).

**Table 1:** Basic characteristics of patients in the three groups ($\overline{x}$ ± s, n = 30)

	Group C	Group RIB	Group RISS	*P*
Age (years)	55.6 ± 11.5	60.5 ± 11.6	58.3 ± 11.5	0.233
BMI (kg/m^2^)	24.3 ± 2.8	23.2 ± 2.9	23.1 ± 2.3	0.143
Procedure duration (min)	93.6 ± 27.6	139.7 ± 35.1	97.4 ± 37.9	0.493
Duration of anesthesia (min)	116.5 ± 25.6	125.1 ± 38.0	118.6 ± 40.1	0.612
ASA class I /II	12 /18	13/17	14/16	0.271
Hypertension	5	9	11	0.212
Diabetes	1	1	2	0.770
Surgical incision (Left chest/right chest)	8/22	10/20	11/19	0.700

Statistical tests: Pairwise comparisons of groups analyzed by one-way ANOVA were made using *post hoc* analyses and the Student–Newman–Keuls *Q*-test

**Fig. 2:**
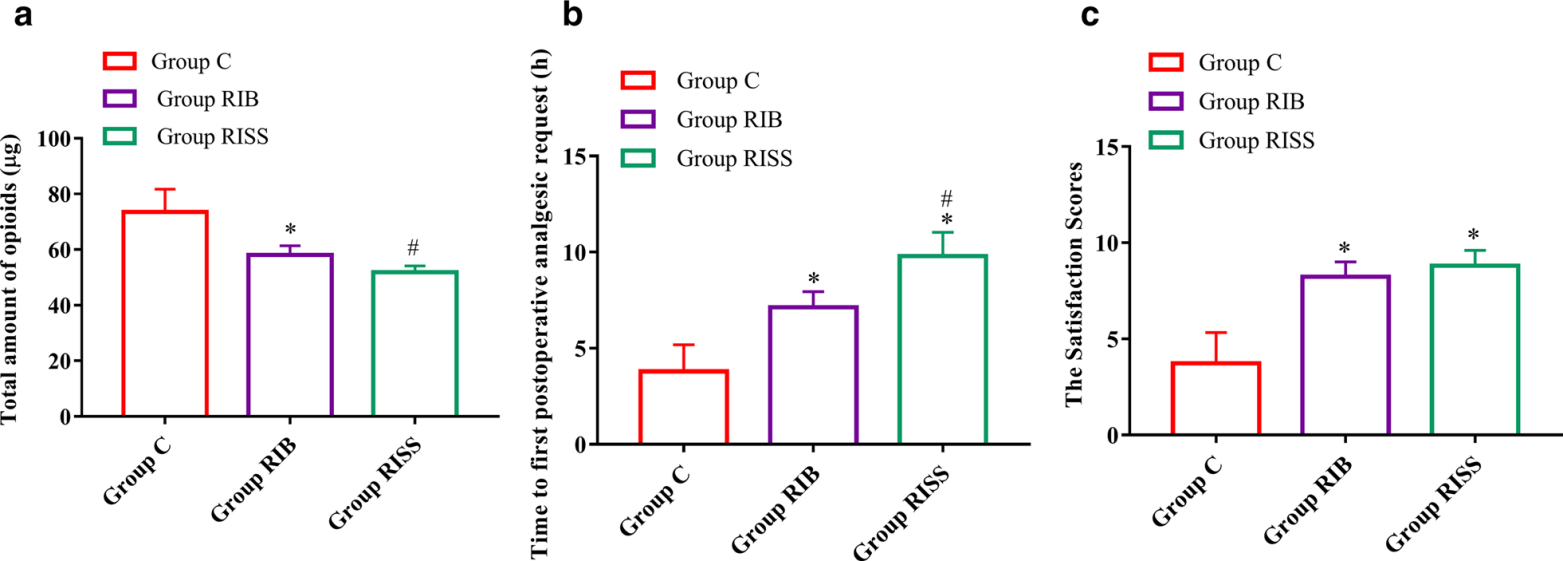
**a** Total opioid consumption (µg) in 24 h; **b** Time to first postoperative analgesic request; **c** Satisfaction score in the 3 groups 24 h after the surgery. *p < 0.05 compared with the control group, ^#^*p* < 0.05 compared with the rhomboid intercostal block combined with sub-serratus plane (RISS) group

**Fig. 3:**
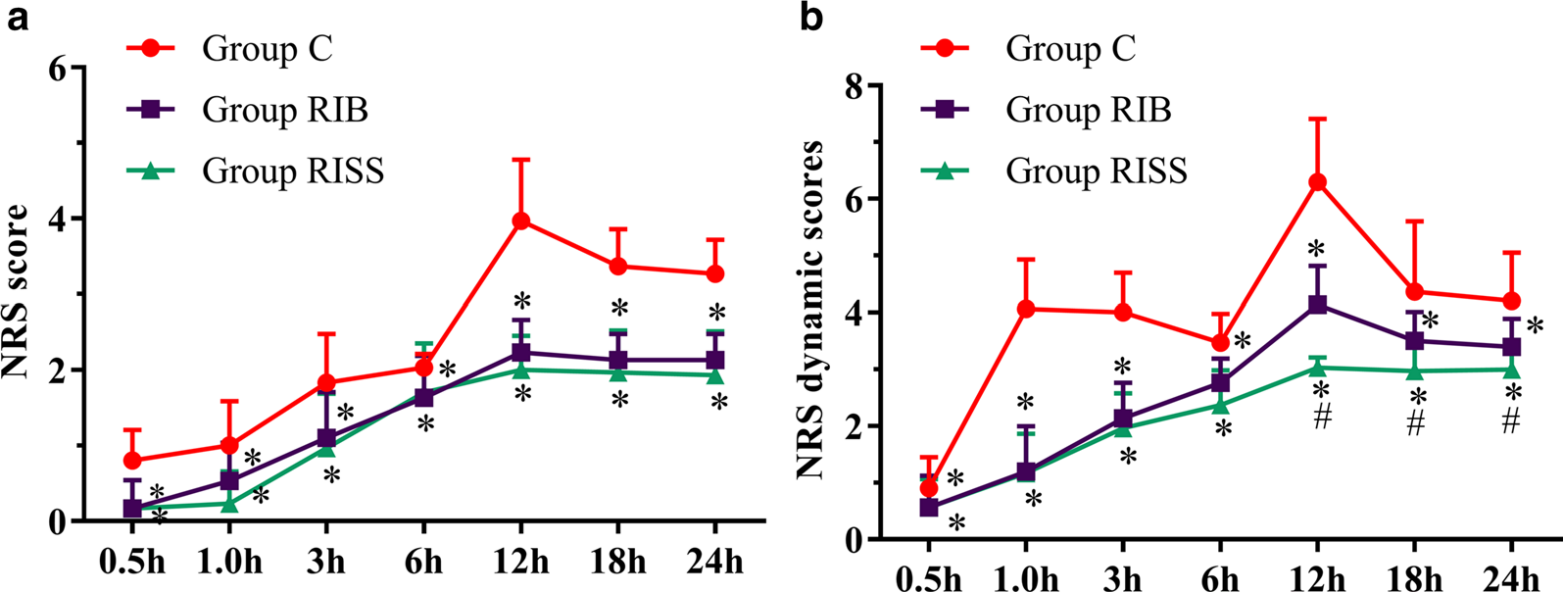
Numerical rating scale (NRS) score at different time points after the surgery in the three groups. **a** NRS scores when the patients were at rest. **b** NRS scores when the patients were active. *p < 0.05 compared with the control group, ^#^*p* < 0.05 compared with the rhomboid intercostal block combined with sub-serratus plane (RISS) group

The dosages of sufentanil consumption at 24 h after the surgery in the RIB and RISS groups were significantly lower than that in group C (58.0 ± 3.4 μg vs. 51.9 ± 2.2 μg vs. 73.5 ± 8.2 μg, *p* < 0.001), and the dosage of sufentanil consumption in the RISS group were also lower than that in the RIB group at 24 h after the surgery (51.9 ± 2.2 μg vs. 58.0 ± 3.4 μg, *p* < 0.001) (Fig. 2). Similarly, the postoperative NRS scores in the RIB and RISS groups at 0.5, 1, 3, 6, 12, 18, and 24 h after the surgery when patients were at rest or active were significantly lower than that in group C (*p* < 0.05 for all comparisons). The NRS scores for the RISS group at 12, 18, and 24 h after the surgery when patients were active were significantly lower than those for the RIB group (*p* < 0.05 for all comparisons) (Fig. 3).

The time to first postoperative analgesic request in the RIB and RISS groups was significantly longer than that in group C (*p* < 0.001 for all comparisons), and the time to first postoperative analgesic request in the RISS group was also longer than that in the RIB group (*p* < 0.001 for all comparisons) (Fig. 2). Compared with group C, the satisfaction scores of patients in the RIB and RISS groups were significantly higher (*p* < 0.05 for all comparisons), but there was no significant difference between the RIB and RISS groups (*p* = 0.054 for all comparisons) (Fig. 3).

There were no significant differences in the dosages of propofol (*p* = 0.450 for all comparisons) and remifentanil (*p* = 0.638 for all comparisons) (Table 2), recovery time (*p* = 0.325 for all comparisons), age (*p* = 0.233 for all comparisons)*,* and BMI (*p* = 0.135 for all comparisons) among the three groups (Tables 1, 2). Additionally, no significant differences were observed in the duration of the surgical procedure (*p* = 0.493 for all comparisons) or duration of anesthesia (*p* = 0.612 for all comparisons) among the three groups (Table 2).

**Table 2:** Intraoperative anesthetic dosage and recovery time in the three groups ($\overline{x}$ ± s, n = 30)

	Group C	Group RIB	Group RISS	F	*P*
Remifentanil (µg)	327.5 ± 120	364 ± 152.23	347.7 ± 170.4	0.452	0.638
Propofol (mg)	315.8 ± 66.9	326.7 ± 110.8	293.7 ± 121.8	0.806	0.450
Recovery time (min)	15.2 ± 1.9	14.6 ± 1.9	14.6 ± 1.4	1.138	0.325

Statistical tests: Pairwise comparisons of groups analyzed by one-way ANOVA were made using post hoc analyses and the Student–Newman–Keuls *Q*-test

The prevalences of postoperative nausea in the RIB and RISS groups were lower than that in group C (*p* = 0.031 for all comparisons). At the same time, the prevalence of postoperative vomiting in the RIB and RISS groups were lower than that in group C (*p* = 0.01) (Table 3). None of the patients reported block-related complications. There were no significant differences in the dosages of propofol (*p* = 0.672 for all comparisons) and remifentanil (*p* = 0.216 for all comparisons), satisfaction scores (*p* = 0.054 for all comparisons), Nausea (*p* = 0.640 for all comparisons)*,* and Vomiting (*p* = 0.554 for all comparisons) between the RIB and RISS group (Table 4).

**Table 3:** Prevalence of adverse events in the three groups (%, n = 30)

	Group C	Group RIB	Group RISS	c^2^	*P*
Nausea (n/%)	10 (33.33%)	3 (10%)	2 (6.67%)	9.128	0.031
Vomiting (n/%)	7(23.33%)	2 (6.67%)	1 (3.33%)	9.120	0.01
Block-related complications (n, %)	0	0	0	–	–

Statistical test: Chi-square test

**Table 4:** Intraoperative anesthetic dosage, satisfaction scores, and prevalence of adverse events in the RIB and RISS group ($\overline{x}$ ± s, %, n = 30)

	Group RIB	Group RISS	c^2^	*P*
Remifentanil (µg)	364 ± 152.23	347.7 ± 170.4	–	0.672
Propofol (mg)	326.7 ± 110.8	293.7 ± 121.8	–	0.216
Satisfaction scores	8.2 ± 0.7	8.8 ± 0.8	–	0.054
Nausea (n/%)	3 (10%)	2 (6.67%)	0.218	0.640
Vomiting (n/%)	2 (6.67%)	1 (3.33%)	0.351	0.554
Block-related complications (n, %)	0	0	–	–

Statistical tests: Pairwise comparisons of groups analyzed by Independent sample *t* test and Chi-square test

## Discussion

This is the first randomized clinical trial to compare the analgesic effects of the RIB and RISS block after VATS. The results of this study showed that Both ultrasound-guided RIB blocker and RISS blocker can effectively reduce the demand for sufentanil within 24 h after VATS, and less sufentanil dosage is needed in patient with RISS blocker. Ultrasound-guided RIB blocker and RISS blocker can effectively relieve pain within 24 h after VATS, and RISS blocker is more effective.

The RIB is a new interfascial plane block described by Elsharkawy et al. [16]. Following the injection of the local anesthetic in the interfascial plane between the rhomboid major and intercostal muscles, the block provides analgesia between the T2 and T9 dermatomes [16]. Besides, the RIB is easy to perform, and the injection site is away from the surgical area. Başak Altıparmak et al. [17] performed ultrasound-guided RIB in two patients for postoperative analgesia after thoracoscopic surgery. They injected 30 ml of 0.25% bupivacaine into the interfascial plane between the rhomboid and intercostal muscles and reported that the NRS scores of the patients were less than 3/10 and that no rescue analgesia was required in the first 12 h. The pin-prick test revealed a sensorial block between T3 and T10 at the postoperative 60th min. Our results are similar to those of Altparmak et al. [17]. In our experiment, we found that after the injection of 20-ml 0.375% ropivacaine with the RIB, the NRS score < 3/10 and the dynamic score ≤ 5/10 within 12 h after the surgery, and no rescue analgesia was required. However, within the postoperative 12–24 h, the analgesic effect of the RIB was poor, and the mean NRS score was approximately 4 in this period.

The RISS plane block is a new interfascial plane block described by Elsharkawy et al. [15]. The authors suggested that the injections of local anesthetics in two tissue planes between the rhomboid and intercostal muscles and deep to the scapula and serratus anterior muscle block the lateral cutaneous branches of the intercostal nerves from T3 to T9. As a result, the RISS block anesthetizes the lateral cutaneous branches of the thoracic intercostal nerves and can be used in multiple clinical settings for chest wall and upper abdominal analgesia [15]. Longo et al. [13] reported the use of the RISS block in non-intubated patients undergoing VATS. They used 15 ml of 0.375% ropivacaine injection in T5–6 and 15 ml of 0.375% ropivacaine injection in T8–9, and at the 1-h and 4-h postoperative evaluation, the patients remained comfortable and pain-free without the need for additional pain medications. Our findings are similar to those of Longo et al. [13]. The RISS block showed better analgesic effects after thoracic surgery, and the NRS scores were maintained at ≤ 3/10 within 24 h after the surgery, and few analgesic remedies were required.

In our study, we also found that compared with the RIB, the RISS block showed longer analgesic effects and achieved lower NRS scores, longer time to first postoperative analgesic request, and fewer complaints of pain. More importantly, patients in whom the RISS block was used required fewer doses of sufentanil than those in whom the RIB was used. We speculate that the reason for the longer duration of analgesia after the RISS block than after the RIB and for the lower NRS pain scores may be the reproducible dermatomal analgesic coverage of the thorax and upper abdomen by the RISS block. Hence, it can be used as a supplement to patch thoracic epidural analgesia [13]. However, there were no significant differences in the intraoperative dosages of propofol and remifentanil, satisfaction scores, and PONV between the two groups.

In current study, 40 ml 0.375% ropivacaine was adminated to the patients with RISS block and 20 ml 0.375% ropivacaine was adminated to the patients with RIB block, no nerve block related complications occurred in all patients. Many previous studies also reported 100–120 ropivacaine for nerve block is safe [18–20]. Ferdinando Longo et al. [18] showed that 35 ml 0.375% ropivacaine was safe and analgesic effective for RISS nerve block. Betul Kozanhan et al. [19] found that 40 ml 0.25% bupivacaine was safe and effective for RISS nerve block. Wei Deng et al. [20] also found that 40 ml 0.375% ropivacaine was safe and analgesic effective for chest wall nerve block.

The following reasons lead to postoperative pain after video-assisted thoracoscopic surgery. First, thoracic drainage tube would cause intercostal neuralgia and pleural stimulation; second, postural changes and severe cough would lead to postoperative pain; third, surgical incision and nerve injury would also lead to postoperative pain. Opioids are usually used to treat postoperative pain, but the incidence of nausea, vomiting is high and the recovery of intestinal function is slow. In current study, we found that the incidence of postoperative nausea and vomiting in patients with no nerve block undergoing thoracic surgery is relatively higher, this may be related to the larger dose of sufentanil used during postoperative. The degree of postoperative pain, the incidence of nausea and vomiting and other postoperative complications may affect patient satisfaction.

Our study has some limitations. We could not evaluate the sensorial block area using a pin-prick test due to the double-blind nature of the study. Second, as the block procedures were performed under general anesthesia, we did not require a sham group in the study. If the patients in the block groups had exhibited back pain related to the injections, they would have been aware of the study groups. Consequently, there would have been a bias in the analysis. However, none of the patients complained of injection pain in the postoperative period. Third, our assessment of the incidence of PONV may not be standard, because we routinely use granisetron as a preventive strategy for PONV.

## Conclusions

Both ultrasound-guided RIB blocker and RISS block can effectively reduce the demand for sufentanil within 24 h after VATS, and less sufentanil dosage is needed in patients with RISS block. Ultrasound guided RIB block and RISS block can effectively relieve pain within 24 h after VATS, and RISS block is more effective.

## Data Availability

Data are available on reasonable request. Data will be available (following de-identification of the participant data) on request via email to the corresponding author (Email:zxy43529@163.com).
