# Correction: Rhomboid intercostal block combined with sub-serratus plane block versus rhomboid intercostal block for postoperative analgesia after video-assisted thoracoscopic surgery: a prospective randomized-controlled trial

**DOI:** 10.1186/s12890-025-03601-4

**Published:** 2025-09-08

**Authors:** Wei Deng, Xiao‑min Hou, Xu‑yan Zhou, Qing‑he Zhou

**Affiliations:** Department of Anesthesiology and Pain Medicine, The Affiliated Hospital of Jiaxing University, Zhejiang Province, Jiaxing, China

**BMC Pulmonary Medicine (2021) 21:68**

10.1186/s12890-021-01432-7

Following publication of the original article, it came to the authors’ attention that there was an error in Table 1: values for duration of anesthesia were erroneously detailed as being shorter than those of procedure duration. The table has since been corrected in the published article; please be referred to the corrected version of Table 1. The authors thank you for reading this erratum and apologize for any inconvenience caused.

**Incorrect Table 1:** Basic characteristics of patients in the three groups ($\overline{x}$ ± s, n = 30)

	Group C	Group RIB	Group RISS	*P*
Age (years)	55.6 ± 11.5	60.5 ± 11.6	58.3 ± 11.5	0.233
BMI (kg/m^2^)	24.3 ± 2.8	23.2 ± 2.9	23.1 ± 2.3	0.143
Procedure duration (min)	141.4 ± 31.2	139.7 ± 35.3	138.8 ± 42.0	0.493
Duration of anesthesia (min)	116.5 ± 25.6	125.1 ± 38.0	118.6 ± 40.1	0.612
ASA class I /II	12/18	13/17	14/16	0.271
Hypertension	5	9	11	0.212
Diabetes	1	1	2	0.770
Surgical incision (Left chest/right chest)	8/22	10/20	11/19	0.700

Statistical tests: Pairwise comparisons of groups analyzed by one-way ANOVA were made using *post hoc* analyses and the Student–Newman–Keuls *Q*-test

**Correct Table 1:** Basic characteristics of patients in the three groups ($\overline{x}$ ± s, n = 30)

	Group C	Group RIB	Group RISS	*P*
Age (years)	55.6 ± 11.5	60.5 ± 11.6	58.3 ± 11.5	0.233
BMI (kg/m^2^)	24.3 ± 2.8	23.2 ± 2.9	23.1 ± 2.3	0.143
Procedure duration (min)	93.6 ± 27.6	103.9 ± 35.1	97.4 ± 37.9	0.493
Duration of anesthesia (min)	116.5 ± 25.6	125.1 ± 38.0	118.6 ± 40.1	0.612
ASA class I /II	12/18	13/17	14/16	0.271
Hypertension	5	9	11	0.212
Diabetes	1	1	2	0.770
Surgical incision (Left chest/right chest)	8/22	10/20	11/19	0.700

Statistical tests: Pairwise comparisons of groups analyzed by one-way ANOVA were made using *post hoc* analyses and the Student–Newman–Keuls *Q*-test

